# Further insight into the global variability of the *OCA2-HERC2* locus for human pigmentation from multiallelic markers

**DOI:** 10.1038/s41598-021-01940-w

**Published:** 2021-11-18

**Authors:** Philippe Suarez, Karine Baumer, Diana Hall

**Affiliations:** Unité de Génétique Forensique, Centre Universitaire Romand de Médecine Légale, Centre Hospitalier Universitaire Vaudois et Université de Lausanne, Lausanne, Switzerland

**Keywords:** Genetics, Molecular biology

## Abstract

The *OCA2-HERC2* locus is responsible for the greatest proportion of eye color variation in humans. Numerous studies extensively described both functional SNPs and associated patterns of variation over this region. The goal of our study is to examine how these haplotype structures and allelic associations vary when highly variable markers such as microsatellites are used. Eleven microsatellites spanning 357 Kb of *OCA2-HERC2* genes are analyzed in 3029 individuals from worldwide populations. We found that several markers display large differences in allele frequency (10% to 35% difference) among Europeans, East Asians and Africans. In Europe, the alleles showing increased frequency can also discriminate individuals with (IrisPlex) predicted blue and brown eyes. Distinct haplotypes are identified around the variants C and T of the functional SNP rs12913832 (associated to blue eyes), with linkage disequilibrium r^2^ values significant up to 237 Kb. The haplotype carrying the allele rs12913832 C has high frequency (76%) in blue eye predicted individuals (30% in brown eye predicted individuals), while the haplotype associated to the allele rs12913832 T is restricted to brown eye predicted individuals. Finally, homozygosity values reach levels of 91% near rs12913832. Odds ratios show values of 4.2, 7.4 and 10.4 for four markers around rs12913832 and 7.1 for their core haplotype. Hence, this study provides an example on the informativeness of multiallelic markers that, despite their current limited potential contribution to forensic eye color prediction, supports the use of microsatellites for identifying causing variants showing similar genetic features and history.

## Introduction

The human eye color trait is the most variable in European populations and it was for a long time considered a simple Mendelian trait with a brown eye color dominant allele and a blue eye color recessive allele^1^. Genome-wide association studies in people of European decent^2,3^ have instead indicated eye color as a polygenic trait^4^ yet characterized by a limited number of major genes. The *OCA2-HERC2* genes explain most of the blue and brown eye color inheritance^5^. Different polymorphisms in the regulatory and coding region of *OCA2* are primarily associated with different eye, hair and skin pigmentation phenotypes^6–9^. These findings increased our understanding of the genetic basis of human pigmentation, and drew attention to their potential applications, such as forensic investigations^10,11^, historical and anthropological researches^12^.

One SNP in particular, rs12913832 in *HERC2*, is responsible for the greatest proportion of eye color predictability, this SNP together with five SNPs located in other genes have been brought together in the IrisPlex eye color prediction panel^11^. The accuracy rate of correctly predicting an individual's eye color as being blue or brown is on average 94% in Europe^13^. Additional variation has yet to be identified to account for the poor success rate for intermediate eye color predictions (73% accuracy) and in admixed populations^14,15^.

The SNP rs12913832 is located in a (11 bp) conserved region of intron 86 of the *HERC2* gene, 21 kb upstream of the promoter of *OCA2*. The rs12913832 C-derived allele is highly associated with European blue eye color as a recessive trait^9^. The ability of this conserved element to act as an enhancer of *OCA2* transcription has been confirmed in experiments using melanocyte cultures carrying either rs12913832 T/T or rs12913832 C/C genotypes^16,17^. The P protein, produced by *OCA2,* is thought to be a mature melanosomal membrane protein, with a potential role in trafficking other proteins to melanosomes^18,19^.

Interestingly, the selection pressure on the *OCA2-HERC2* region associated with blue eye color in Europeans has been strong^19,20^. This region encompass the third longest haplotype spam of diminished heterozygosity in the genome of modern Europeans^21^ which implies intense selection at this locus in ancestral European populations. Multiple factors possibly played a role such as sexual^22^, the ability to overcome seasonal affective disorder^23–25^ and associated light skin increased risk for developing melanoma and nonmelanoma skin cancer^26^. Several lines of research indicate that selective pressure for light pigmentation acted independently in Europeans and East Asians, yet with some genes in common. The brown-eyed associated SNPs frequent in Europeans are different from that of Asians, suggesting a population specific history of the genetic component of pigmentation^19,27^.

Evidences from haplotype analysis comparing Dutch and Mediterranean population samples suggest that blue eye color has only arisen once during the Neolithic period, past 6–10,000 years ago, as a founder mutation shared by diverse European populations^17^. During the great agriculture migration to the northern part of Europe, the mutations spread out from the Black Sea region. Newer studies have also indicated that some *OCA2* missense mutations give rise to blue eye color, when the genotype rs12913832 C/T predicts brown eye color and that these are only found in the Scandinavian population and not in individuals of Southern-European descent^6,28^. The *OCA2-HERC2* is therefore a region of large interest due to its functional role and population genetics. Global SNP variation has been comprehensively described, yet more data are being available from ongoing projects of whole genome sequencing which indicate that multiallelic sites represent as much as 10% of the genomic variants.

In this study, we aim at further investigating the genetic variability of *OCA2-HERC2* by using microsatellite markers (STRs) as an example of multiallelic variants. We are interested in exploring how the use of polymorphisms with a different mutational mechanism and higher rate, change the pattern of haplotype structures and allelic associations around functional alleles. Conveniently, the recent history of selection of *OCA2* should enhance the degree of correlations of neutral variants even when highly variable polymorphisms are used. Our interest in these data is twofold: on the one hand, to provide an empirical example of the utility of STRs in studies of association mapping of similar traits, on the other hand, to search for rs12913832 proxy STR markers for a presumptive eye color DNA test to be included in conventional forensic STR multiplexes.

The density of genetic markers required for successful association mapping of complex diseases depends on linkage disequilibrium between non-functional markers and functional variants. There are few reports about the pattern or extent of linkage disequilibrium (LD) between SNPs and STRs genomewide^29–31^. Yet, several results suggest that association studies using not only SNPs but also multiallelic STRs within or near candidate loci would be useful to search for a disease susceptibility gene, especially in populations with unknown LD structure. The rationale behind this is that in non-African samples highly significant LD between microsatellite alleles and stable markers is preserved across relatively large genetic distances (about 100 Kb range)^29^ compared to the shorter range (about 30 Kb) for SNPs^32^. The average length of LD for microsatellites is ~ 100 Kb, which is considerably higher than that of SNPs^33,34^. Therefore, a single microsatellite captures a larger genomic region than does a single SNP^35^. One STR can harbor both types of alleles: those showing complete association with a SNP and alleles that show little or no association. STR outside LD blocks may still show LD with SNPs inside the LD block. In that case, for association studies we would not need an STR in each haplotype block as for SNPs. At the same time, microsatellites show a smaller interpolation variability^36,37^ and a single-step expansion or contraction of the tandem repeat on the background of ancestral SNP haplotypes can break up common haplotypes, leading to greater haplotype diversity within the linkage disequilibrium block of interest. The relative performance of STR and SNP in association mapping will also depend on the frequency of disease variants. Markers alleles achieve maximal power for detecting associations when disease alleles are at similar frequencies^38^. As a result, STR have the potential to find rare disease variants that common SNPs will miss^39,40^. Several genome wide association studies used tens of thousands of STRs to investigate the genetic basis of hypertension, narcolepsy, anorexia nervosa, mandibular prognathism, rheumatoid arthritis, type 2 diabetes and prostate cancer^41–47^.The known susceptibility gene for rheumatoid arthritis *HLA-DRB1* was successfully confirmed and two new candidates (*TNXB* and *NOTCH4*)^43^ were identified through a combination of STR and SNP analysis. Microsatellites have starred in association studies leading to widely replicated discoveries of type 2 diabetes (*TCF7L2*)^46^ and prostate cancer genes (the 8q region)^45^. Moreover, the ability of microsatellite markers to capture information on coding SNPs was successfully tested for the human major histocompatibility complex to predict HLA functional SNPs^48,49^.

However, patterns of LD between SNPs and microsatellites markers may vary considerably between loci^50^. To increase the accuracy of power studies in STR based disease mapping it is therefore important to provide empirical data for genomic regions of large interest and well characterized as the *OCA2-HERC2* locus.

Here we present our results on the global variation of eleven microsatellites spanning 357 Kb in the *OCA2-HERC2* region and their correlation with the functional SNP rs12913832 and the eye color trait as predicted by the IrisPlex assay. We also examine degrees of LD, related microsatellites’ haplotype structures and homozygosity levels.

## Results

### STR markers variability

**Figure 1:**
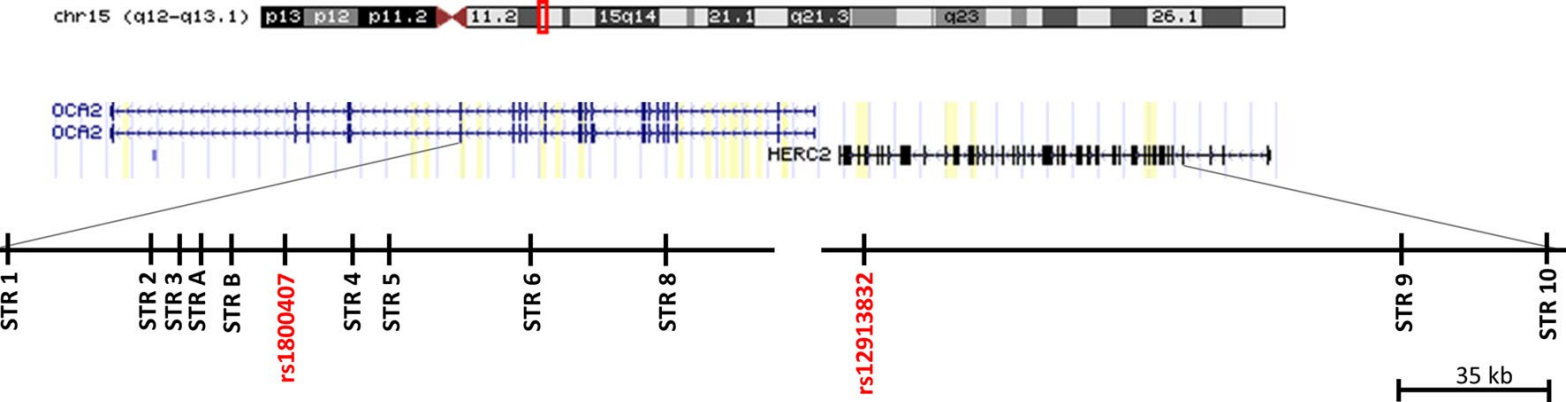
Position of STR markers included in this study relative to intron–exon structure of *OCA2-HERC2* genes on chromosome 15 q12-q13.1. Two key SNPs for eye color prediction, rs1800407 and rs12913832 of the IrisPlex panel are also included.

A total of 11 microsatellites polymorphisms are the focus on this study. They span the region from intron 18 of *OCA2* to intron 5 of *HERC2*. Seven STRs are dinucleotide repeats, three are tetranucleotide and one is a pentanucleotide repeat sequence. Markers’ distances and number of alleles are indicated in Fig. 1 and Table 1.

**Table 1:** STR marker list.

Name	GRCh38/hg38	Repeat	Distance^a^ (bp)	N alleles
STR 1	27′922′681	GT	31′436	17
STR 2	27′954′117	CA	6′474	13
STR 3*	27′960′591	TCTA	5′371	6
STR A	27′965′962	GTTT	6′866	6
STR B	27′972′828	ATTTT	12′344	9
rs1800407	27′985′172		16′087	
STR 4*	28′001′259	CA	8′426	3
STR 5	28′009′685	CA	32′959	17
STR 6	28′042′644	TAAA	8′641	5
STR 8	28′074′004	CA	46′468	5
rs12913832	28′120′472		125′095	
STR 9	28′245′567	CA	34′049	18
STR 10	28′279′616	CA		19

^a^Position from UCSC Genome Browser on Human Dec. 2013 (GRCh38/hg38).

*Low level of polymorphism, one allele at frequency between 94 and 100% outside Africa.

### Allele frequency distribution in diverse human populations

**Figure 2:**
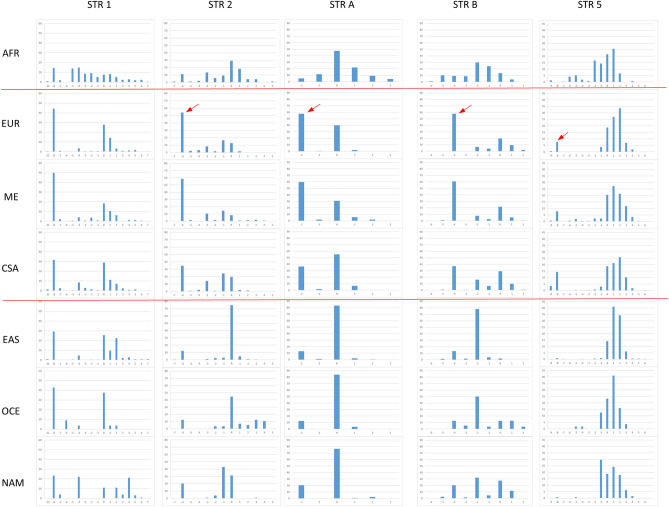

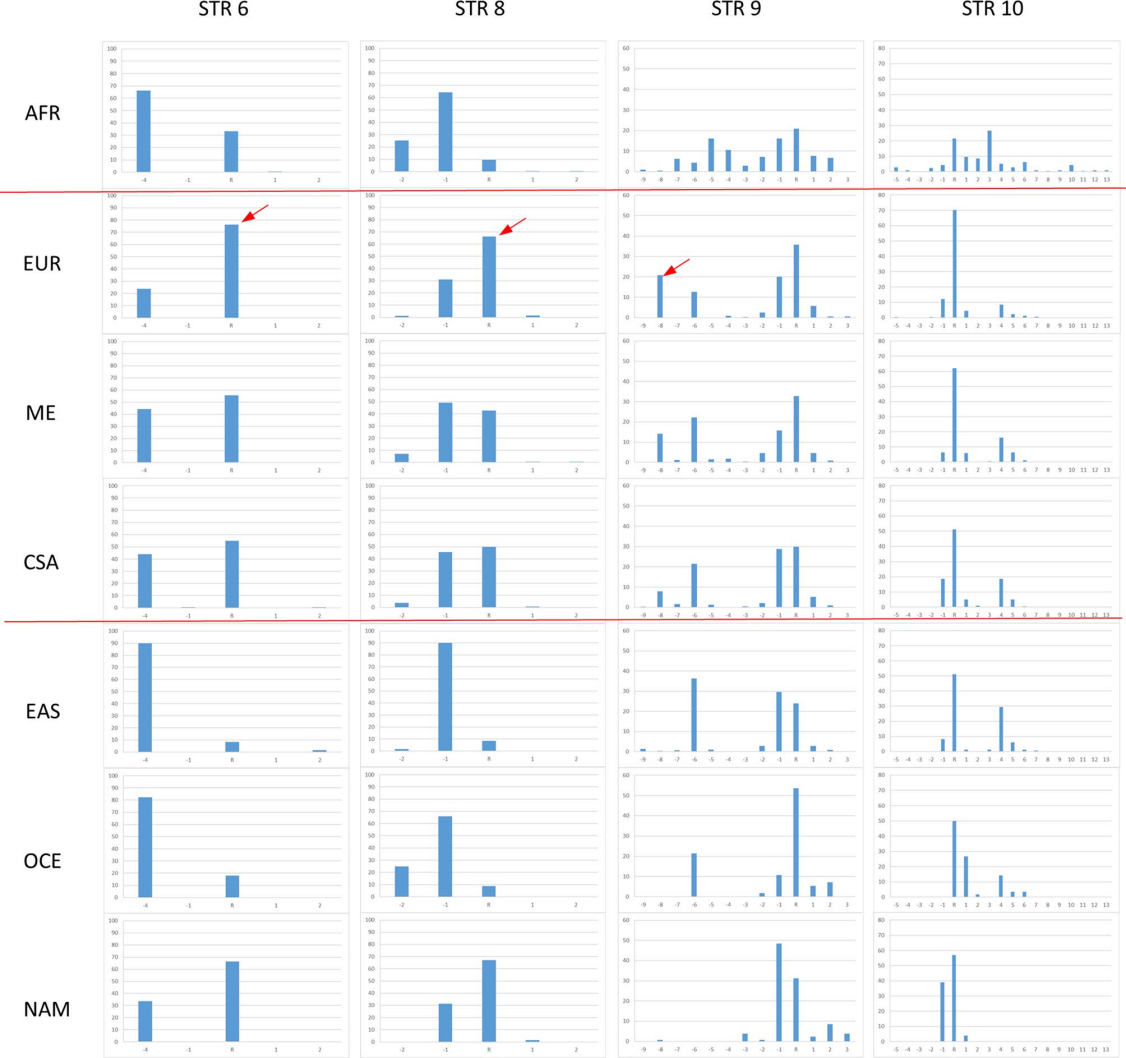
Allele frequency distributions of nine STR markers estimated for the major HGDP-CEPH population groups of Africa (AFR n = 105), Europe (EUR n = 158), Middle East (ME n = 162), Central-South Asia (CSA n = 202), East Asian (EAS n = 230), Oceania (OCE n = 28) and Native America (NAM n = 64). Each bar indicates the frequency value of the allele named in the x-axis. Low polymorphic STR 3 and 4 are not reported. Red arrows indicate alleles highly frequent (about 50–60%) or private to Eurasia and low frequent (about 10%) outside this geographic region. Two horizontal lines separate Eurasian populations from the others.

Figure 2 shows markers’ allele frequencies for the HGDP-CEPH populations grouped according to the seven major geographic regions Africa, Europe, Middle East, Central-South Asia, East Asia, Oceania and Native Americans. Two markers are not reported here because of the low genetic diversity, these are STR 3 and STR 4. They show one major allele at frequency between 94 and 100% outside Africa.

Results of Fig. 2 are consistent with other studies of genetic variability of multiallelic markers: (1) Africa appears as the most variable region with the largest number of alleles also showing similar frequency values. (2) Patterns of allele frequencies are similar among Eurasians groups: Europe, Middle-East and Central-South Asia, and different from those observed for East Asians, Oceanians and Native-Americans. Conversely, peculiar of this study, is the presence of one allele highly frequent (about 50–60%) in Eurasians and low frequent (about 10%) outside this region across several markers (red arrows). This is true for the allele − 6 of STR 2, − 2 of STR A, − 4 of STR B. Alleles − 8 of STR 5 and − 8 of STR 9, are not highly frequent but almost private to Eurasians. The allele R of STR 6 and R of STR 8 show also high frequency in Native-Americans besides Eurasians.

### Allele frequency distribution in Europeans with predicted blue and brown eye colors

The HGDP-CEPH European group includes 158 individuals. Prediction of eye color is possible using the IrisPlex marker set available for all DNA samples^11^. Based on these data we selected individuals of Europe with a probability of blue or brown eye color higher or equal to 0.8, this arbitrary cut-off was used to reduce the error rate associated to such indirect determination of the phenotype, together with eliminating all individuals with predicted “intermediate eye color”. This analysis determined two groups, 42 predicted blue eyes and 62 predicted brown eyes individuals. In the HGDP-CEPH collection, outside Europe, only two individuals of Central-South Asia and three of Middle-East had a value of blue eye color prediction higher or equal to 0.8. To avoid a population structure bias we limited our analysis to Europeans.

**Figure 3:**
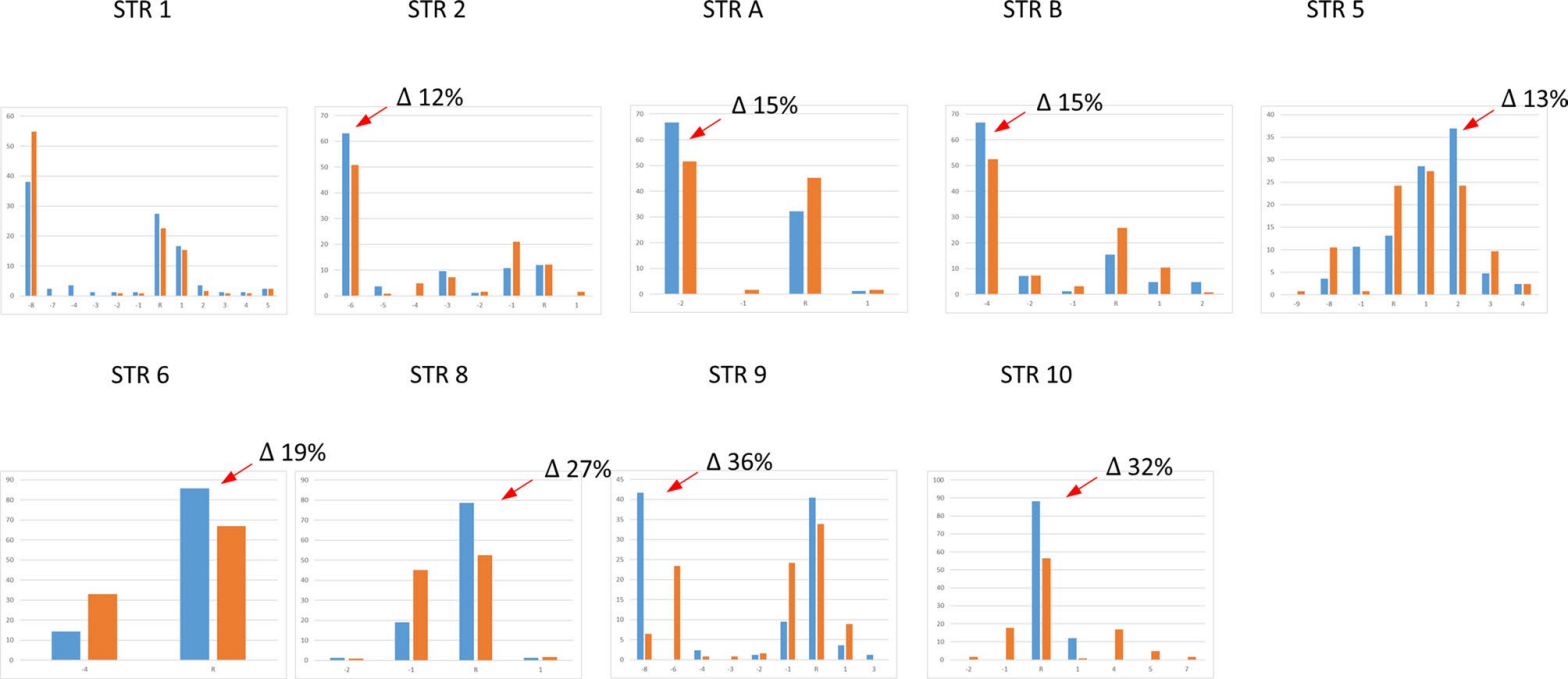
Allele frequency distributions of nine STR markers estimated for predicted brown eye (n = 62) color Europeans (brown bar) and predicted blue eye (n = 42) color Europeans (blue bar). Red arrows indicate the alleles with increased frequency in blue eye color predicted individuals (differences in frequency are indicated by Δ n%).

**Figure 4:**
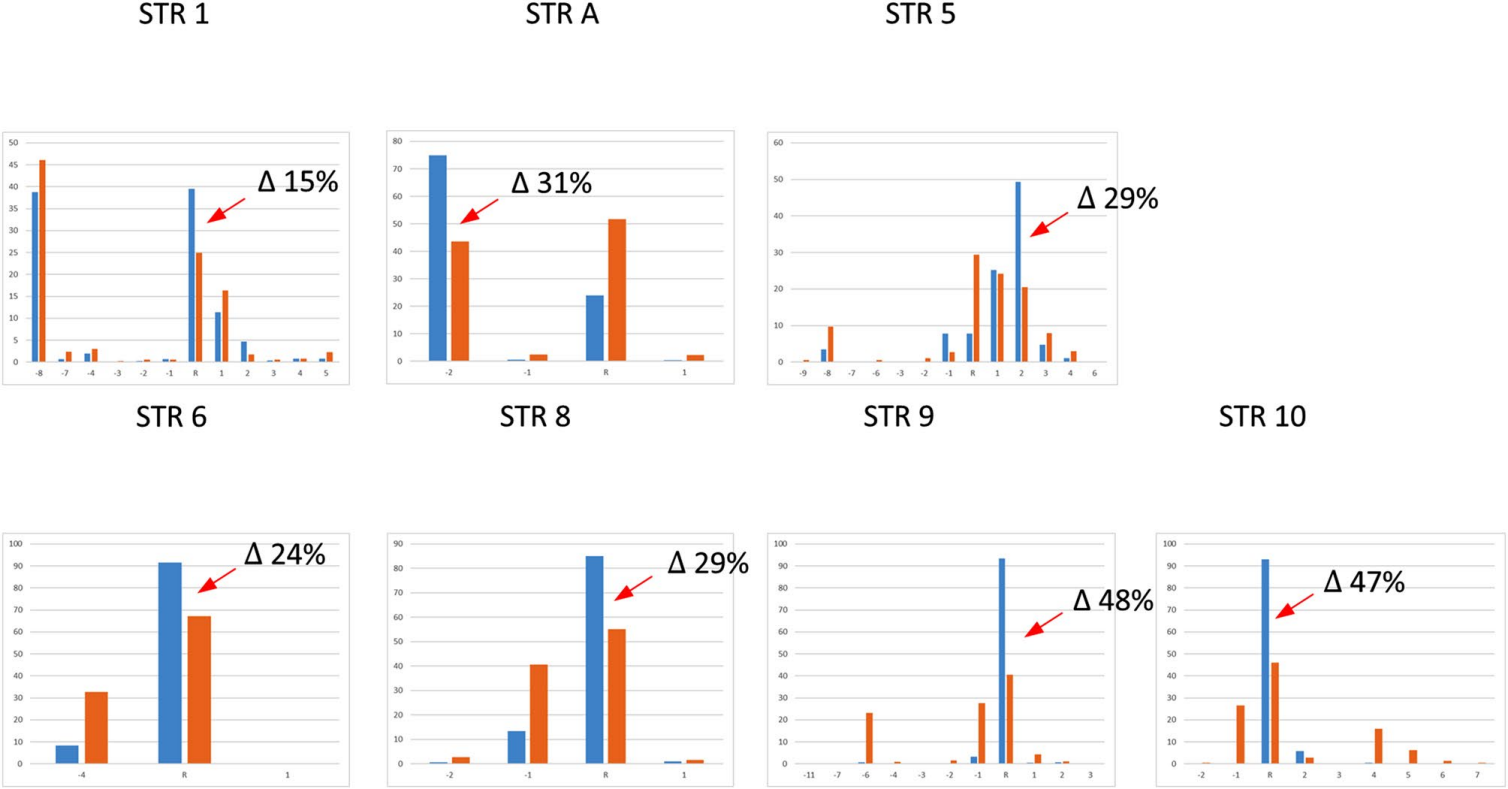
Allele frequency distributions of seven STR markers in Europe estimated for the haplotypes containing the functional allele rs12913832 C (blue bar, n = 1105 European haplotypes) and for haplotypes containing the alternative variant rs12913832 T (brown bar, n = 641 European haplotypes). Red arrows indicate the alleles with increased frequency in the group of haplotypes carrying the functional variant for blue eye color (differences in frequency are indicated by Δ n%). STR 2 and STR B are not reported because of the large proportion of missing data in the gnomAD database.

When STRs’ allele frequencies are compared between predicted brown-eyed versus blue-eyed Europeans (Fig. 3) several alleles show increased frequency values ranging from 12 to 36% increase in predicted blue eye color individuals (red arrows). Most of the time these alleles are the same that also showed marked differences across major population groups. The analysis of a larger group of Europeans (n = 876) using also data available from gnomAD (see methods) shows a similar pattern of allele frequency distribution (data not shown). STR 2 and STR B could not be included because of more than 50% of missing data in the database. To further explore the relationship of the functional SNP rs12913832 and surrounding STRs, we compared single STR allele frequencies between haplotypes containing the alleles C, determinant for blue eye color (blue bar, n = 1105 European haplotypes), to those containing the alternative variant T (brown bar, n = 641 European haplotypes) (Fig. 4). The observed differences are similar to those of Fig. 3 yet, values are higher going from 14 to 48% especially around rs12913832. Note that the allele -8 of STR 9 is undistinguishable from the allele R according to the sequencing data of genomAD (see “Methods”).

The statistical significance of the differences in allele frequency distribution of the data reported in Fig. 3 and 4 was assessed by Chi-square tests and Monte Carlo simulations (Table 2). We report both the results from our genotyped HGDP-CEPH European samples on the right, and the results from the larger sample set from the gnomAD database parsed based on the functional SNP rs12913832 on the left. In both sample sets STR 5, 6, 8, 9 and 10 showed marked differences with *P* values ranging from 10^−3^ to < 10^−6^ including the haplotype of STR 6–8–9–10 *P* value 10^−6^ (data not shown). Smaller *P* values ranging from 10^−5^ to < 10^−6^ are obtained for the same STRs in addition to STR A for the larger dataset when rs12913832 C and T containing haplotypes are compared.

**Table 2:** Significance of allele frequency differences between predicted blue and brown eye color HGDP-CEPH Europeans and between European haplotypes containing rs12913832 C and T.

Marker	Blue (n = 42) and brown (n = 62) eye color		rs12913832 C (n = 1105) and T (n = 641) haplotypes	
	*P* value (T1)	*P* value (T2)	*P* value (T1)	*P* value (T2)
STR 1	0.2	0.07	0.59	0.27
STR 2	0.03	0.12	–	–
STR A	0.05	0.03	0.00003	0.00001
STR B	0.07	0.1	–	–
STR 5	0.003	0.009	0.000005	0.00003
STR 6	0.002	0.003	0.000008	0.000007
STR 8	0.0002	0.0001	0.00002	0.00001
STR 9	< 10^−6^	< 10^−6^	< 10^−6^	< 10^−6^
STR 10	< 10^−6^	< 10^−6^	< 10^−6^	< 10^−6^

The significance of allele frequency differences between predicted blue and brown eye color individuals was estimated by Monte Carlo approaches using the program ‘clump’^61^.

T1: Normal chi-square with significance assessed by Monte Carlo simulations.

T2: Chi-square from table after collapsing columns with small expected values together and significance assessed by Monte Carlo simulations.

### Haplotypes and linkage disequilibrium values

**Figure 5:**
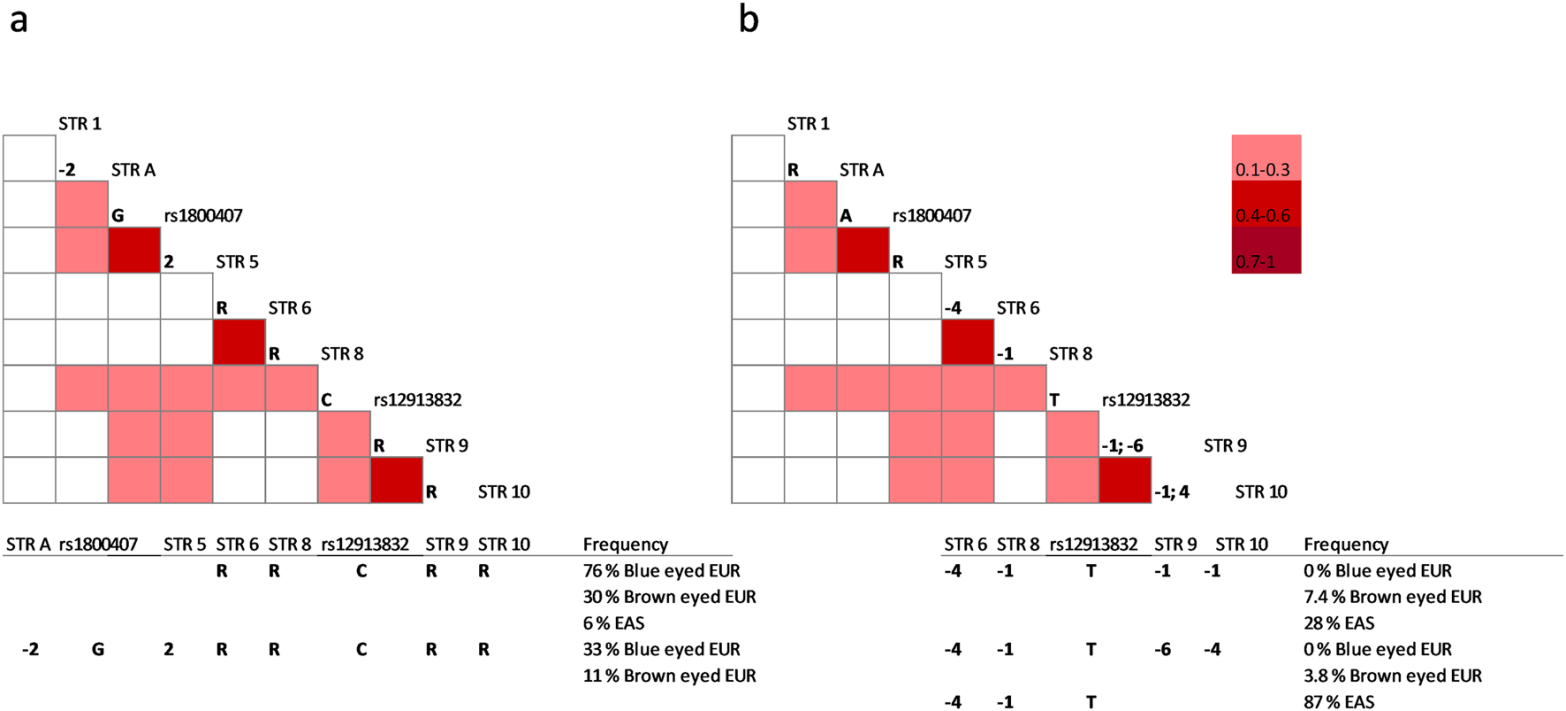
European haplotype block structure and pattern of LD of the *OCA2-HERC2* region including selected polymorphic STR markers and SNPs rs1800407 and rs12913832. LD r^2^ values are showed by the standard color scheme indicated. Only values higher or equal to 0.1 associated to a minimum of five allele counts are reported. (**a**) indicates allelic associations around the allele C of rs12913832 (n = 1105), (**b**) indicates allelic associations around the allele T of rs12913832 (n = 641). Below the plots are reported selected haplotypes and relative frequency values in specific populations.

Significant linkage disequilibrium r^2^ values for Europeans are shown in Fig. 5 by color-coded graphics. Two graphics (a, b) are shown to report alternative multiallelic haplotypes with associated alleles. The C allele of rs12913832, is associated to neighboring alleles and forms a core haplotype including STR 6–8–9–10 (RRRR) (Fig. 5a). This haplotype is highly frequent in predicted blue eyed Europeans 76% and less frequent in predicted brown eyed Europeans 30% and very low 6% in East Asians. The composing alleles all appeared more common in Europe and in predicted blue eye color individuals. This haplotype is 237 Kb long and encompasses two LD blocs previously described. The frequency of the larger haplotype (313.6 kb) from STR A to STR 10 (-2G2RRRR) is still highly frequent in predicted blue eyed Europeans (33%) while its frequency is only 11% in predicted brown eyed individuals.

The alternative allele T of rs12913832, is part of two different haplotypes (-4-1-1-1) and (-4-1-6-4) (Fig. 5b) that are 7.4% and 3.8% frequent in predicted brown eye color Europeans and absent in predicted blue eyed individuals. The haplotype (-4-1-1-1) is 28% frequent in East Asians and the shorter haplotype -4-1-T from STR 6 to rs12913832 in the same population is 87% frequent. Another haplotype around rs1800407 is highly frequent (67%) in East Asia (data from HGDP-CEPH collection only), this includes alleles (RR-2) of STRs 2, A, B (LD plot not shown). STR2 is 2′226 bp distant from rs1800414, the SNP most associated to human pigmentation in Asia. In Europe, its frequency goes down to 5% with no differences between predicted blue and brown eye colors. In East Asia, high frequency haplotypes do not show allelic associations based on r^2^ values (Supplementary Fig. 1).

### Homozygosity levels and allelic associations

Homozygosity values of STR markers in predicted blue eye Europeans are higher than in predicted brown eye individuals and increase for markers located around rs12913832, with values reaching 75% to 91% (Table 3). As before, eye color phenotype is predicted using the IrisPlex marker genotypes available in gnomAD for the large European dataset of 876 individuals. For the allele showing increased frequency in predicted blue eye individuals, odds ratios reach values of 4.2, 7.4 and 10.4 for the four STRs around rs12913832 and 7.1 for the core haplotype RRRR.

**Table 3:** Homozygosity and allelic associations in predicted blue and brown eye color Europeans.

Marker	% Homozygosity		Positive allele	Frequency		OR	95% CI	*P* value
	Blue eyed	Brown eyed		Blue eyed	Brown eyed			
STR 1	33	35	R	0.409	0.263	1.9	1.56–2.49	< 0.0001
STR 2	–	–	–	–	–	–	–	–
STR A	59	45	− 2	0.740	0.553	2.3	1.8–2.9	< 0.0001
STR B	–	–	–	–	–	–	–	–
STR 5	31	27	2	0.490	0.283	2.4	1.93–3.05	< 0.0001
STR 6	84	56	R	0.907	0.697	4.25	3.13–5.75	< 0.0001
STR 8	75	44	R	0.848	0.573	4.2	3.2–5.4	< 0.0001
STR 9	91	45	R	0.936	0.553	8	5.9–10.99	< 0.0001
STR 10	88	45	R	0.935	0.581	10.4	7.42–14.63	< 0.0001
STR 6–8–9–10	59	21	RRRR	0.756	0.305	7.1	5.46–9.15	< 0.0001

Predicted blue eyed individuals n = 361 ; predicted brown eyed individuals n = 312. Odds ratios (OR) 95%, confidence interval (CI) and *P* values were calculated using the software MedCalc.

### Haplotype association

Next we explored whether homozygous individuals for the core haplotype RRRR of STR 6–8–9–10 are all predicted blue eyed color similarly to homozygous individuals for rs12913832 C. Table 4 shows that indeed most of the homozygous RRRR/RRRR are predicted blue eyed (n = 167) and a very minor group is instead predicted brown eyed (n = 18). Also, most of the individuals homozygous for any other haplotype ----/---- are predicted brown eyed (n = 136) and few (n = 17) blue eyed. For these two genotypes the haplotype RRRR is a good proxy for the genotype of rs12913832 yet heterozygous individuals with one copy of RRRR are not all predicted brown eyed as rs12913832 would indicate, instead almost equally distributed between the two phenotypes (n = 109 and n = 138).

**Table 4:** The core haplotype RRRR of STR 6–8–9–10 in predicted blue and brown eye Europeans compared to rs12913832 genotypes in the same individuals.

Marker	Genotype	Blue eyed	Brown eyed
STR 6–8–9–10	RRRR/RRRR	167	18
	RRRR/----	109	138
	----/----	17	136
rs12913832	C/C	361	0
	C/T	0	139
	T/T	0	173

---- indicates any haplotype not RRRR.

## Discussion

This study focuses on the most important genomic region for eye color variation in humans, which is *OCA2-HERC2*. Numerous studies based on biallelic (SNP) polymorphisms, previously elucidated functional variants and patterns of allelic associations including signatures of natural selection in Europe. Here we further investigate this region with a set of microsatellites annotated from whole genome sequencing data. With respect to previous studies based on SNP data, this type of polymorphism (STR) should provide non-redundant genetic information due to the different mutational mechanisms and its effect on patterns of allelic associations and haplotype structure. Here we show for the first time the correlation between multiallelic markers and functional variants of the eye color phenotype.

Eleven microsatellites spanning 357 Kb across the *OCA2-HERC2* locus were genotyped in 1,064 individuals from 52 populations around the world (HGDP–CEPH panel). Data available from whole genome sequencing projects (gnomAD) were retrieved for the same STR when possible, this allowed us to work with larger sample collections from Europe (n = 876) East Asia (n = 801) and Africa (n = 896). Haplotypes were reconstructed integrating the two SNPs of the IrisPlex assay that are located on this chromosome, rs1800407 and rs12913832 and the eye color phenotype was predicted using the 6 SNP panel of IrisPlex.

The results showed that nine STRs were polymorphic across major population groups. Allele frequency distributions showed as expected, Africa as the most variable region. Europe, Middle-East and Central-South Asia form an undistinguishable homogeneous Eurasian group different from East Asians, Oceanians and Amerindians.

Interestingly, large differences in allele frequency distributions (up to 36%) were found between predicted blue and brown eyed individuals of Europe with often one allele showing increased frequency in predicted blue eye color. It should be noted here that the actual phenotype data of the individuals was not available and the IrisPlex method was reported to have lower prediction accuracy for intermediate eye colors (non-blue and non-brown) and heavily relies on rs12913832^51^. Although, only samples showing a prediction value for blue or brown higher or equal to 0.8 were included, the phenotyping procedure may have an error rate estimated to be 3–4%^11,28^. Because of this limitation, the subsequent analyses of allele imbalance and LD using the larger population data from gnomAD rather focused on the correlation between STRs and the functional variant rs12913832.

Differences in frequency values go up to 48% when considering two groups of haplotypes containing the variants C or T of rs12913832 in 876 Europeans, with markers STR 5, 6, 8, 9, 10 showing statistical significance. LD analysis indicated some degree of allelic associations among SNPs and STRs, these are more abundant in Europe, where the associated alleles are those that showed skewed allele frequencies between predicted blue and brown eye individuals. Previous studies also showed large difference in SNP haplotypes frequencies between blue and brown eye color individuals going from 36^52^ to 54%^9^ including four SNPs in a region from STR 4 (17 Kb upstream) to STR 9 (22.6 Kb downstream) and to rs12913832, respectively.

Two haplotype blocks were identified from STR A to rs1800407 and from STR 6 to STR 10. Linkage disequilibrium r^2^ values are significant up to 295 Kb from rs1800407 to STR 10 and values range from 0.1 to 0.5. Note that r^2^ values higher than 0.1 should allow identifying a disease susceptibility variation in an association study^35,53^. This block includes the functional variant rs12913832, three different haplotypes are associated to the allele C and T of rs12913832, one is most frequent in predicted blue eye individuals 76% (30% in blue) and two others exclusive to predicted brown eye individuals with 7.3% and 4% frequency. Finally, homozygosity values reach levels of 75% to 91% around rs12913832 in predicted blue eye individuals, which are consistent with the recessive mode of inheritance of the blue eye color. In addition, elevated odds ratios values up to 8 and 10.4 were obtained around rs12913832 and for the core haplotype RRRR of STR 6–8–9–10.

Previous studies using SNPs genotyped in a large population of Netherland indicated three LD blocks going from the position corresponding to STR 2 to rs1800407 for 27 kb (LD1), around STR 6 for 13 Kb (LD2) and around STR 9 for 70.5 kb (LD3)^52^. These blocks are also detected by this STR based study with some allelic associations going beyond originally defined blocks LD2 and LD3 up to 237 Kb. Eiberg and colleagues^17^ described a 175 kb long haplotype of 13 SNPs, 97% frequent in Danish individuals with blue eye color. The corresponding position of this block with respect to our data is 27 Kb upstream rs12913832 to 22.5 Kb downstream STR 9.

This study designed around known causal variants confirms that allelic associations with functional SNPs can be detected over greater distance with STRs than with SNPs^33,34^. This is because the higher mutation rates of STRs underlie a stronger statistical significance of LD. Therefore the importance of using STR also for the first screen of association study to reduce the number of initial association tests or when LD blocks are not known. Although many studies support these predictions theoretically, few have attempted to provide empirical data on the extent of LD detectable at STR and SNP sites at defined distance flanking unknown functional variants of significant effect. In the context of alcohol dependence syndrome studies, the persistence of allele association and differences in allele frequencies of a range of STR markers was investigated around the functional locus aldehyde dehydrogenase *ALDH2* known to be under selective constraint in Japanese alcoholic populations^33^. This study showed the persistence of LD over distances up to 400 Kb for pairs of loci including at least one STR. It follows that the comparison of allele frequency differences for the STR markers in the case (alcoholics) and control populations would have detected the *ALDH2* marker as a putative susceptibility locus. It should be noted that the recent origin of the *ALDH2* functional markers is very likely to be a major factor determining the strength of the association observed. With regard to the observation of the allele frequency pattern difference between the cases and controls, population demography, allele frequencies and degree of natural selection are additional important factors in determining the success of gene hunting using this type of approach. Similarly, the genetic influence of *IL10* SNP allele on HIV-1 infection and AIDS progression was first deduced by observations of epidemiology associations of two STR loci within 4 Kb of the *IL10* gene^54^. In the same way, the data presented here show that considering the two extremes of brown and blue predicted eye color (most robust prediction by IrisPlex) and *OCA2-HERC2* as candidate genes, simple parameters of skewed allele frequencies of STRs and levels of homozygosity would allow us to point out the most relevant genetic region carrying the functional variant rs12913832, even with a relatively small sample size and a degree (3–4%) of mislabeled phenotype due to the indirect ascertainment.

In addition, these data confirm the different genetic history of eye color in Asians that showed no allelic associations around rs12913832 and one frequent haplotype that is rare in Europe. The patterns of variability we observed also support previous data indicating strong natural selection in this genomic region in Europe.

## Conclusions

This study further describe novel SNP-STR correlations spanning a region of large interest for human genetics, population genetics and forensic science. These results show that in this region of *OCA2-HERC2*, markers mutating more rapidly than SNPs also capture the effect of demographic history and selective pressure by showing marked genetic differences between predicted blue and brown eyed individuals within the same population. These data support the hypothesis that with STR markers, besides SNPs, it is possible to investigate the genetics of eye color and effectively use this type of variation as marker for mapping causing variants of similar traits, appeared recently, with large penetrance and under positive selection.

Models for detecting variants responsible of genetic traits have been poorly constrained by available data leading to large uncertainties in model predictions. Studies like our go toward the effort of providing a portion of such empirical results. We hope that these data can help validating novel statistical methods aiming at using multiallelic markers for detecting functional variants.

Finally, the idea of possibility replacing functional SNP genotyping with linked STRs for roughly predicting the eye color phenotype in Europe, is poorly supported by the data. A small set of STRs could be easily added to current forensic DNA profiling multiplex, yet none of the STRs analyzed is strongly associated to rs12913832 to work as proxy. This is not surprising since the highest OR observed with these STR data is about 10 while a single SNP in *HERC2* previously showed OR of about 30^55^, due to the difference in ORs and therefore effects sizes of markers, the prediction using STR markers would be lower than using functional SNPs. Conversely, STRs alleles around rs12913832, are not as randomly distributed as one could expect from highly variable loci. In particular, for individuals found to be homozygous RRRR/RRRR around rs12913832 C/C the blue eye color would be predicted in 90% of the cases. Similarly, individuals that lack the haplotype RRRR around rs12913832 T/T are predicted brown eyed color in 89% of the times. Unfortunately, no indications of the phenotype can be obtained from all the other combinations of heterozygous haplotypes. Finally, there is great potential in investigating STRs in the *HERC2-OCA2* region to explain the eye color that is wrongly predicted by rs11913832. These data may provide the basis of such future studies that should rely on precise and direct eye color phenotype determination.

## Methods

All methods were performed in accordance with the relevant guidelines and regulations of the journal.

### Population samples for genotyping

The CEPH Human Genome Diversity panel (HGDP-CEPH) contains 1,064 individuals from African, European, North African/Middle Eastern, Central-South Asian, East Asian, Native American and Oceanian populations^56^. For all data analyses purposes we considered only 952 individuals (H952 subset) after exclusion of duplicates, first- and second-degree relatives^57^. Populations were combined into continental-based groups which have been previously established^58^ with the following composite populations, sample sizes and labels: 6 African (n = 105 AFR), 8 European (n = 158 EUR), 4 North African/Middle Eastern (n = 162 ME), 9 Central-South Asian (n = 202 CSA), 17 East Asian (n = 230 EAS), 2 Oceanian (n = 28 OCE) and 5 Native American (n = 64 NAM). A second population sample included 84 unrelated European individuals with Caucasian appearance and Swiss parents since three generations. DNA was extracted using the QIAamp DNA Mini kit (Qiagen AG Switzerland) according to the manufacturer’s guidelines and quantified using the Quantifiler Human DNA Quantification Kit (Life Technologies).

For all subjects, blood cell samples were obtained according to protocols and informed-consent procedures approved by the institutional review board *Commission cantonale d'éthique de la recherche sur l'être humain (CER-VD)*, and were labelled with an anonymous code number linked only to demographic information and sex.

### Available genotypes from genome sequencing projects

The genotypes of additional individuals were obtained from the Genome Aggregation Database (gnomAD v3.1.1 variants, https://gnomad.broadinstitute.org/downloads) ^59^. The gnomAD v3.1.1 track shows variants from 143,150 unrelated individuals sequenced as part of various population-genetic and disease-specific studies, some markers have only frequency values while others show single individual genotypes. All variants are mapped to the GRCh38/hg38 reference sequence. From this database, markers STR 2 and STR B showed low quality genotypes with more than 50% of missing data. The population samples with individual genotypes from gnomAD used in this study include 634 Europeans (Finnish ‘fin’ and Non-Finnish European ‘nfe’), 791 Africans and 571 East-Asians. In summary, the largest sample collections considered in this study include 876 individuals from Europe, 896 form Africa and 801 from East-Asia.

### Marker selection and typing

STR markers were identified by searching the *OCA2-HERC2* genomic region for simple tandem repeats located by Tandem Repeats Finder^60^. DNA samples were genotyped for the 11 STRs selected to overlap the region from SNP rs2703969 to rs1667394, where most of linkage and association studies for eye color reported positive results. PCR reactions were performed in 20 μl final volume. This contained 1 × PCR Buffer containing 1.5 mM MgCl_2_ (Thermo Fisher), 125 μM dNTP (Thermo Fisher), 1.2 U AmpliTaq Gold DNA Polymerase (Thermo Fisher) and 0.5 ng DNA. Primers’ sequences, quantities and multiplexes are indicated in Supplementary Table S1. PCR thermal cycling conditions were: 5 min at 95 °C, 1 min at 94 °C, 1 min 55 °C, 1 min at 72 °C for 30 PCR cycles and a final extension of 30 min at 72 °C.

PCR fragments were separated by capillary electrophoresis after adding 1 μl PCR amplicon to 8.5 μl deionized formamide HI-DI (Thermo Fisher) and to 0.5 μl 600 LIZ size standard (Thermo Fisher). Capillary electrophoresis was performed using an ABI PRISM 3130xl Genetic Analyzer (Thermo Fisher) according to the manufacturer's instruction and analyzed using the GeneMapper® ID v3.2.1 software (Thermo Fisher), with a minimum peak height threshold of 50 RFU. The commercial DNA CEPH 1347-02 (Thermo Fisher) was added to two empty positions in each PCR plate as positive control of amplification and internal standard for allele calls, at least one empty well per plate was used a negative control of amplification.

Markers were grouped in three multiplexes as indicated in the table. For allele names, ‘R’ indicates the number of repeats of the reference genome and additional repeats are indicated by ‘+ n’ or’-n’ consistent with gnomAD. Considering all populations, 723 HGDP-CEPH samples genotypes are also reported by gnomAD. These data were used for confirmation and harmonization of allele calls. All the genotypes produced here corresponded to the data available in gnomAD except for STR 9 where we could distinguish the alleles -8 and R, while these two are the same allele R in gnomAD. The allele − 8 was observed only in the HGDP-CEPH Eurasian populations. This is probably due to a neighboring deletion linked to the allele R coamplified by the PCR primers used in this study. It is also possible that, because of the complexity of the tandem repeat sequence, that includes both variable CA repeats and variable TA repeats, two different neighboring sites instead of one are indicated in gnomAD.

The eye color prediction for the 84 Swiss Europeans samples was done by IrisPlex typing according to the protocol described in Walsh et al. 2011^11^ and by using the model from the online tool (https://hirisplex.erasmusmc.nl/). The IrisPlex consists of 6 SNPs, rs12913832 (HERC2), rs1800407 (OCA2), rs12896399 (SLC24A4), rs16891982 (SLC45A2/MATP), rs1393350 (TYR) and rs12203592 (IRF4).

As previously published, the protocol consists of a single multiplex two step PCR using 1 µl genomic DNA extract (concentration of 0.5 ng/µl) and primers in a 12 µl reaction which includes 1 × PCR buffer, 2.7 mM MgCl_2_, 200 µM of each dNTP and uses adjusted thermocycling conditions for increased specificity: (1) 95 °C for 10 min, (2) 33 cycles of 95 °C for 30 s and 61 °C for 30 s, (3) 5 min at 61 °C. Each primers concentration used was 0.208 µM except for rs1800407 used at 0.104 µM. This was followed by product purification (2.5 µl of PCR product) using an Exo I / SAP treatment (NEB) in CutSmart 10 × Buffer for an incubation time of 90 min at 37 °C and inactivation step of 15 min at 80 °C. Further multiplex single base extension (SBE) reaction using the ABI Prism1 SNaPshot kit (Applied Biosystems) was performed, 1 µl of purified product, SNaPShot reaction mix and Sequencing Primer mix were used in a final volume of 5 µl. Thermocycling conditions used were: (1) 96 °C for 2 min, (2) 25 cycles of 96 °C for 10 s and 50 °C for 5 s, (3) 30 s at 60 °C. The SNaPShot PCR product was then treated with SAP (NEB) for 90 min at 37 °C and inactivation for 15 min at 80 °C. One microliter of cleaned products was analyzed on the ABI 3130xl Genetic Analyser (Applied Biosystems) with POP-4 on a 36 cm capillary length array. Run parameters were optimized to increase sensitivity, with an injection voltage of 6 kV for 11 s, and run time of 1000 s at 60 °C.

### Data analysis

Allele frequencies were estimated by gene counting. The statistical significance of the difference in allele frequency between predicted blue and brown eye color individuals was assessed with the CLUMP program^61^, which implements a Monte Carlo approach by performing repeated simulations.

The EM algorithm was used to estimate maximum likelihood haplotype frequencies by using Arlequin version 3.5.1.2^62^ as well as pairwise linkage disequilibrium *r*^2^ values. Odds ratios (OR) 95% confidence interval (CI) and *P* values of Table 2 were calculated using the MedCalc software.

### Ethics approval

The current study was approved by the Centre Hospitalier Universitaire Vaudois and Université de Lausanne institutional review board. Genomic DNA samples fully-consenting individuals were collected by the Human Genome Diversity Project (HGDP), in a collaboration with the Centre Etude Polymorphism Humain (CEPH) in Paris. For all subjects, blood cell samples were obtained according to protocols and informed-consent procedures approved by institutional review boards, and were labelled with an anonymous code number linked only to demographic information and sex. Besides the HGDP-CEPH diversity panel human cell line samples, all other samples involved in the study are long lasting anonymized DNA extracts previously obtained with informed written consent from healthy individuals for research purposes.

### Consent to participate

Each blood sample used was freely donated under conditions of informed consent to participate.

### Consent for publication

Each blood sample used was freely donated under conditions of informed consent to publish.

## Supplementary Information

Supplementary Figure 1.

Supplementary Table S1.

## Data Availability

Markers information and genotypes will be available right after publication acceptance at the HGDP-CEPH database (http://www.cephb.fr/en/hgdp_panel.php#basedonnees).
