# Supplementary material for: Further insight into the global variability of the OCA2-HERC2 locus for human pigmentation from multiallelic markers
Source: Sci Rep. 2021 Nov 18;11:22530. doi: 10.1038/s41598-021-01940-w (PMC8602267; doi:10.1038/s41598-021-01940-w)
Supplement: Supplementary file 1 — Supplementary Figure 1. [file 41598_2021_1940_MOESM1_ESM.pdf]

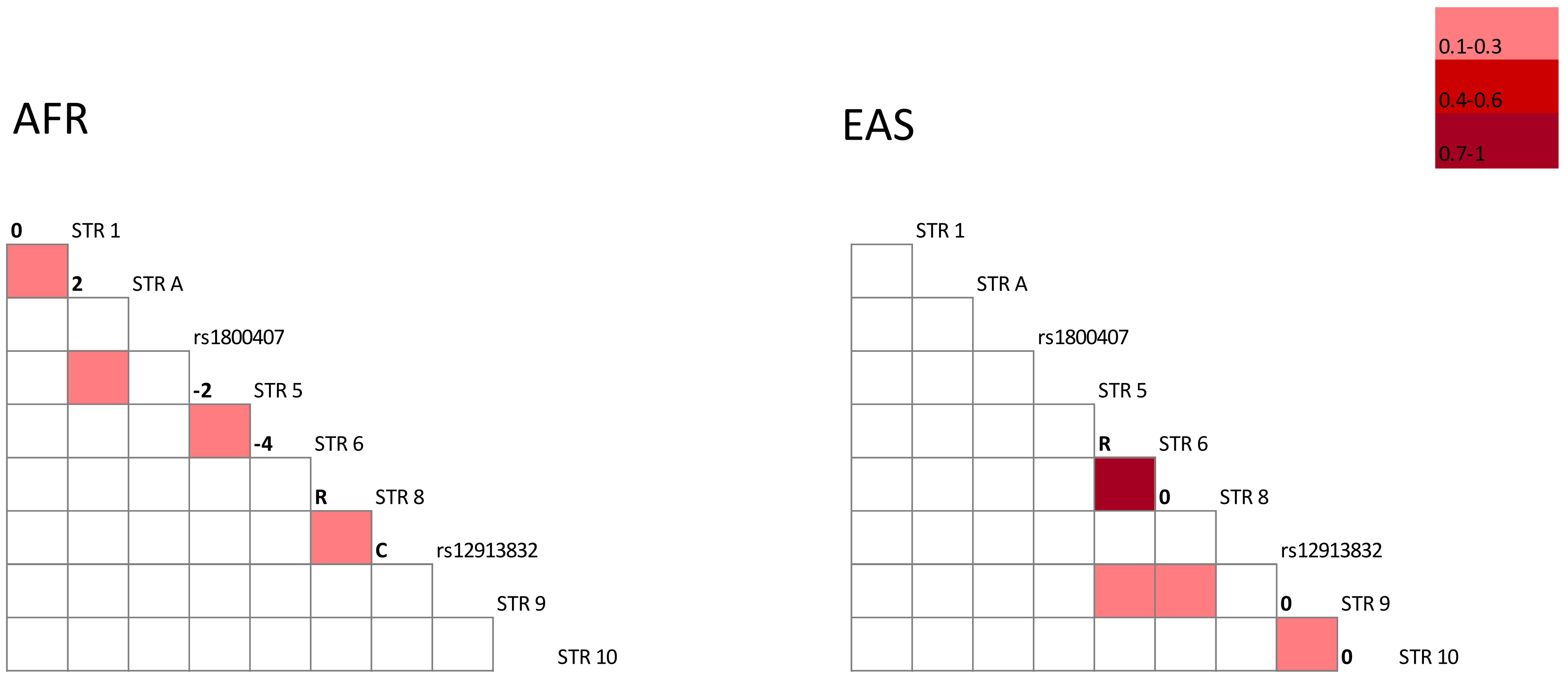

Supplementary Fig. 1 Haplotype block structure and pattern of LD in Africans (AFR n=896) and East Asians (EAS n=801). LD r^2 values are showed by the standard color scheme indicated. Only values higher or equal to 0.1 associated to a minimum of five allele counts are reported.
