# Supplementary material for: Further insight into the global variability of the OCA2-HERC2 locus for human pigmentation from multiallelic markers
Source: Sci Rep. 2021 Nov 18;11:22530. doi: 10.1038/s41598-021-01940-w (PMC8602267; doi:10.1038/s41598-021-01940-w)
Supplement: Supplementary file 2 — Supplementary Table S1. [file 41598_2021_1940_MOESM2_ESM.pdf]

Table S1

PCR primers.

STR	Primer forward seq 5’-3’	Primer reverse seq 5’-3’	Concentration (nM)	Multiplex
STR 1	ATTTCCCTTGGCTGTGAATG	GCACATGGGAGAAACAAACA	15	2
STR 2	ACTGGTCACCAAGTCCATCC	GCTTGGCAACATCCCTGTAT	5	2
STR 3	GCTATAAACCTGCCCCTGCT	CAAGCAGCTGAGATTACTGGTG	305	3
STR A	AACGATAAGATGGCCCTAAGC	CCCTGTCAATCAAGAGCACA	75	1
STR B	TTTCTTGGCCAGTTGCATATC	CCAGCTTGGGTGAGAAAGAG	200	1
STR 4	AGATATCTGTGCGGCCATGT	TTAGCATTCAGGTTCGTCCA	305	2
STR 5	GGGAATCACTTGAACCCACA	TCCCTGAGACTTCCACTTTGA	10	2
STR 6	CTCCAGCCTAGGTGGCATAG	GCATCTTGGTTTGGCAATTC	5	3
STR 8	AGGGTTGTGTGAGGGATTTCT	TTGGCTATTCGATGCCACTT	3.5	3
STR 9	GGGCAACAGAGCAAGATGTAG	TGGAGATAATCCAAATCAGAAGAA	15	3
STR 10	CTGAGAGGCTGAAGCAGGAA	CCTTGAACTCCAGGGCTCTA	3.5	3
